# Development of a Mosquito Repellent Formulation Based on Nanostructured Lipid Carriers

**DOI:** 10.3389/fphar.2021.760682

**Published:** 2021-10-11

**Authors:** Daniele Carvalho Abrantes, Carolina Barbara Rogerio, Jhones L. de Oliveira, Estefânia V. R. Campos, Daniele Ribeiro de Araújo, Laurindo Cesar Pampana, Marcelo José Duarte, Geórgio Freesz Valadares, Leonardo Fernandes Fraceto

**Affiliations:** ^1^ Institute of Science and Technology, São Paulo State University (UNESP), São Paulo, Brazil; ^2^ Faculty of Agronomy and Veterinary Sciences, São Paulo State University (UNESP), São Paulo, Brazil; ^3^ Federal University of ABC, São Paulo, Brazil; ^4^ Veterinary Products Institute, Araçoiaba da Serra, Brazil

**Keywords:** Aedes aegypti, repellent, arboviruses, nanoparticles, nanostructure lipid carriers

## Abstract

Arboviral diseases are a threat to global public health systems, with recent data suggesting that around 40% of the world’s population is at risk of contracting arboviruses. The use of mosquito repellents is an appropriate strategy to avoid humans coming into contact with vectors transmitting these viruses. However, the cost associated with daily applications of repellents can make their use unfeasible for the low-income populations that most need protection. Therefore, the development of effective formulations offers a way to expand access to this means of individual protection. Consequently, research efforts have focused on formulations with smaller quantities of active agents and sustained release technology, aiming to reduce re-applications, toxicity, and cost. The present study investigates the development of nanostructured lipid carriers (NLCs) loaded with a mixture of the compounds icaridin (synthetic) and geraniol (natural), incorporated in cellulose hydrogel. The NLCs were prepared by the emulsion/solvent evaporation method and were submitted to physicochemical characterization as a function of time (at 0, 15, 30, and 60 days). The prepared system presented an average particle size of 252 ± 5 nm, with encapsulation efficiency of 99% for both of the active compounds. The stability profile revealed that the change of particle size was not significant (*p* > 0.05), indicating high stability of the system. Rheological characterization of the gels containing NLCs showed that all formulations presented pseudoplastic and thixotropic behavior, providing satisfactory spreadability and long shelf life. Morphological analysis using atomic force microscopy (AFM) revealed the presence of spherical nanoparticles (252 ± 5 nm) in the cellulose gel matrix. Permeation assays showed low fluxes of the active agents through a Strat-M^®^ membrane, with low permeability coefficients, indicating that the repellents would be retained on the surface to which they are applied, rather than permeating the tissue. These findings open perspectives for the use of hybrid formulations consisting of gels containing nanoparticles that incorporate repellents effective against arthropod-borne virus. These systems could potentially provide improvements considering the issues of effectiveness, toxicity, and safety.

## Introduction

Arthropod-borne virus (arboviruses) are a global public health concern since most of them are related to neglected tropical diseases. Several arthropod can transmit arboviruses to human, including dengue, chikungunya, Zika, yellow fever among others (Guarner and Hale, 2019). Although *Aedes aegypti* is the most popularly known vector, arboviruses can also be transmitted to humans by ticks, midges, flies, and fleas. Arboviral diseases are a global problem for public health systems, with recent data suggesting that 40% of the human population is at risk of such diseases (Leta et al., 2018). Hence, the development of ways to prevent contact between the mosquito and humans continues to be one of the strategies strongly recommended by national and international health protection agencies. Among these methods, insect repellents are used as the first line of defense against mosquito bites and arbovirus transmission (CDC, 2019; Ministério da Saúde, 2019). However, the cost of daily repellent applications may be unfeasible for low-income populations that most need protection. Therefore, the development of effective and inexpensive formulations is one way to expand access to this method of individual protection.

The synthetic repellents currently most widely used against *Aedes aegypti* are DEET (considered as the gold standard), icaridin (KB3023), and IR3535. Icaridin (ICA) is a promising repellent derived from pepper, which was developed in the 1990s and began to be used in 1998, and has been shown to be effective in protecting against insect bites (Naucke et al., 2007). However, there are concerns about the biological impacts of these compounds and their potential toxicity towards nontarget organisms (Webb and Hess, 2016). Another option is to use botanical repellents such as the essential oil of citronella grass (*Cymbopogon nardus*), which has high contents of geraniol and citronellal. However, the duration of its protection is very short, compared to synthetic repellents, due to its high volatility, and direct application to the skin is not recommended, due to possible irritation (Maia and Moore, 2011; Rehman et al., 2014). Therefore, the development of formulations that include suitable carrier systems can contribute to the use of such compounds.

An ideal repellent formulation should provide low permeation through the skin; a pleasant odor; prolonged repellent action; broad activity against insects, especially the main vectors of interest; agreeable aesthetic and sensory characteristics; resistance to water and perspiration; high chemical stability; no staining of fabrics and clothing; and low cost (Tavares et al., 2018). Generally, higher concentrations of active agents provide longer-lasting effects (Kuri-Morales et al., 2017), but they can cause local toxicity, in addition to increasing costs. A promising solution is to use formulations containing repellents encapsulated in nanoparticles, which can decrease the dosages required and prolong the duration of release of active agents, consequently reducing toxicity and the number of applications required (Tavares et al., 2018). Furthermore, the formulations can become more affordable, due to the smaller amounts of active agents used.

Studies have reported the encapsulation of botanical repellents in different controlled release systems, resulting in positive effects such as prolonged action and low permeation (Chang and Dobashi, 2003; Maia and Moore, 2011; Solomon et al., 2011; Misni et al., 2016; Lammari et al., 2020). Neem oil, citronella, and icaridin, encapsulated separately in nanoparticles, have recently been marketed by a company supplying high-performance products (NanoScoping, 2020a; 2020b). Previously, another similar company encapsulated 5.5% icaridin in lipid nanoparticles (Nanovetores, 2017). Up to now, there have been no commercial formulations based on nanoparticles containing a mixture of botanical and synthetic agents, and there have been no studies in the literature concerning this type of formulation. Therefore, in the present work, attention was focused on the synthesis of repellents containing a mixture of icaridin and geraniol. These compounds belong to different chemical classes, suggesting that the spectrum of action could be enhanced, with possible synergistic effects, as observed in studies of the larvicidal effects of botanical extracts in combination with synthetic insecticides (Shaalan et al., 2005; Raghavendra et al., 2013). This could enable the development of an innovative product for use as a repellent.

In the last 2 decades, nanostructured lipid carriers (NLCs) have emerged as innovative systems for topical and transdermal applications (Sharma et al., 2017). These materials were developed as a second generation of lipid nanoparticles, with a composition consisting of a mixture of solid and liquid (oil) lipids. The solubility of active agents is usually much higher in oils than in solid lipids, enabling higher loading capacity and prolongation of the release of active agents (Fang et al., 2008). Various approaches have been used to improve performance in dermal and transdermal applications. The pharmaceuticals and cosmetics industry has employed a wide range of NLCs, due to their ease of preparation, feasibility of scale-up, biocompatibility, and prolonged release of the encapsulated substances (Doktorovova and Souto, 2009; Junyaprasert et al., 2009; Khosa et al., 2018).

The majority of cosmetics and topical medications exhibit viscoelastic behavior, which facilitates application and spreadability. For this purpose, hydrogels are widely used as gelling agents in the formulations (Binder et al., 2019). Hydroxypropylmethylcellulose (HPMC), a hydrophilic polymer derived from cellulose, has the characteristic of forming chemical interactions between the chains, creating a three-dimensional network. It is frequently used in biomedical applications, due to its biodegradability and biocompatibility. In low concentrations of around 2%, it can be used to form a film at the application site, enhancing the local persistence of the active agent (Desfrançois et al., 2018; Hay et al., 2018).

Therefore, the aim of this work was to develop, characterize, and evaluate the applicability of repellent formulations based on geraniol (GRL) and icaridin (ICA) encapsulated in nanostructured lipid carriers (NLCs) in suspension or incorporated in HPMC hydrogel. The NLCs were characterized in terms of their size distribution, polydispersity index (PDI), zeta potential (ZP), and encapsulation efficiency. Analyses of the nanocarriers were performed using differential scanning calorimetry (DSC), atomic force microscopy (AFM), and physicochemical stability measurements. Release assays, *in vitro* permeation tests, and accelerated stability studies were also performed. The system was shown to be a potential repellent that could contribute to reducing diseases caused by arboviruses transmitted by *Aedes aegypti*.

## Materials and Methods

### Materials

Icaridin 97% (ICA) was obtained from Chemidin (China). Geraniol 98% (GRL) was obtained from Quinarí (Ponta Grossa, Paraná, Brazil). Tripalmitin, polyvinyl alcohol (PVA), and hydroxypropylmethylcellulose (HPMC) were obtained from Sigma-Aldrich (St. Louis, United State). Myrytol was obtained from BASF. The ethanol used was from Labsynth (*Diadema*, São Paulo, Brazil). Acetonitrile (HPLC grade) was from J.T. Baker (United State). Other reagents were obtained from local suppliers in Sorocaba (São Paulo, Brazil).

### Preparation of the Nanostructured Lipid Carriers

The NLCs were prepared by the emulsion/solvent evaporation method (Vitorino et al., 2011). The organic phase was composed of lipids (myritol and tripalmitin) and the active agents (ICA and GRL), diluted in chloroform. This phase was mechanically mixed (using a Turrax homogenizer at 14,000 rpm) with the aqueous phase composed of 1.25% PVA (polyvinyl alcohol) surfactant and ethyl alcohol. The solvent was evaporated until the formulation reached a final volume of 10 ml, resulting in active agent concentrations of 5% (w/v) for ICA and 2% (w/v) for GRL. As a control, empty NLCs were synthesized using the same procedure, but without addition of the active agents in the organic phase. The emulsion was used as a control in specific comparative analyses. Due to the low solubilities of the repellent chemicals in water, the mixture of 5% ICA +2% GRL was emulsified using 1.25% PVA, the same concentration used in preparation of the nanoparticles.

### Incorporation of the Lipid Carriers in Hydrogel

Incorporation of the lipid carriers in HPMC was performed after synthesis of the nanostructured lipid carriers loaded with 5% ICA and 2% GRL, and the empty NLCs. For this, 2% of HPMC powder was slowly added to the suspension of NLCs, with homogenization for 10 min (Turrax, 10,000 rpm). A control gel was prepared similarly, dissolving 2% of HPMC powder in distilled water.

### Physicochemical Characterization of the Nanostructured Lipid Carriers in Suspension

Size distribution and polydispersity index (PDI) measurements were performed using the dynamic light scattering (DLS) technique. Zeta potential determination was performed by microelectrophoresis. These analyses employed a ZetaSizer Nano ZS90 system (Malvern Instruments, United Kingdom) operated with a fixed angle of 90°, at 25°C, with the samples diluted 200 times. In addition, measurements of nanoparticle concentration, size distribution, and polydispersity were performed by nanoparticle tracking analysis, with the samples diluted 20,000 times, employing a NanoSight instrument (Malvern Instruments, United Kingdom) equipped with a green laser (532 nm) and a sCMOS camera controlled by NanoSight v.3 software. The results were obtained as the average of three analyses. The formulations were kept at ambient temperature, with evaluation of their stability over time (at 0, 15, 30, 60, 90, and 120 days). The encapsulation efficiency was determined using the microcentrifugation technique, with quantification of the non-encapsulated active compounds in the filtrate by high performance liquid chromatography (HPLC), using an Ultimate 3,000 instrument (Thermo Fisher Scientific, Waltham, United State), with Chromeleon 7.2 software for acquisition and analysis of the chromatograms. The mobile phase used for analysis of icaridin was methanol:water (65:35 v/v), while acetonitrile:water (60:40 v/v) was used for geraniol, in both cases employing a Phenomenex Gemini C18 column (150 × 4.60 mm, 5 µm). The tests were performed in triplicate. Linear regression applied to the points of the calibration curves resulted in the following equations: y = 4.97732x + 0.04689 (GRL, Supplementary Figure S2) and y = 0.26518 x—0.01767 (ICA, Supplementary Figure S3).

### Morphology of the Nanostructured Lipid Carriers in Suspension and in the Gel

The morphology and size distribution of the nanoparticles were analyzed by atomic force microscopy (AFM), using a Nanosurf Easyscan 2 Basic BT02217 instrument. The suspension of nanoparticles was diluted 20,000 times, deposited on silicon plates, and placed in a desiccator. In the case of the nanoparticles incorporated in gel, a smear of the formulation was used, without dilution. The images (256 × 256 pixels, TIFF format) were obtained using a TapA1-G probe (BudgetSensors, Izgrev, Bulgaria) in tapping mode at 90 Hz. The images were processed using Gwyddion software (Dublin, Ireland).

### Differential Scanning Calorimetry

The differential scanning calorimetry technique was used to investigate the interactions among the components of the nanoparticle formulations and the hydrogel matrix. The calorimetric curves were obtained using a Q20 system (TA Instruments), with heating from 0 to 250°C, at 10°C/min, under a flow of nitrogen at 50 ml/min. The results were presented as thermograms showing the melting temperature of a reference material, compared to the studied sample.

### Mechanical Properties: Rheological Analysis

The rheological properties of the formulations were analyzed using an oscillatory rheometer (Kinexus lab, Malvern Instruments, United Kingdom) with cone and plate geometry (20 mm diameter, 0.5 rad angle, and 1 mm gap). The repellent formulations (1 g) were placed in the sample holder and evaluated at skin temperature (32.0 ± 0.5°C) and using a temperature ramp (from 10 to 50°C, at 5°C/min), at a frequency of 1 Hz and shear stress of 1 Pa. The aim was to determine whether the rheological properties changed after application on the skin or during shelf storage. The samples were carefully applied to the lower plate of the rheometer and allowed to equilibrate for 1 min, prior to analysis. In addition, oscillatory analyses were performed with frequency scanning in the range 0.1–10 Hz, with shear stress of 1 Pa and constant temperature of 32.5°C. Continuous rheological measurements were performed using shear rates in the range 0.01–500 s^−1^, for both the ascending and descending curves, with each step having duration of 120 s.

### Determination of the Release Profiles and Mechanisms of Release of the Repellents From the Formulations

Investigation of the *in vitro* release profiles for the liquid and gel NLCs formulations employed a vertical diffusion system with a regenerated cellulose membrane (MWCO 12000–14000 pores) coupled to two compartments (donor and receptor). The system was operated under sink conditions. A volume of 100 ml of 3% PVA was used as co-eluent in the receptor solution. The system was kept in a closed environment, at a controlled temperature of 32.5°C, under constant magnetic stirring during the assay (Iikura et al., 2019). The NLC formulations (5% ICA +2% GRL) were applied (1 g of gel, or 1 ml of suspension) in the donor compartment, after which 1 ml aliquots were periodically removed from the receptor compartment, replacing the volume removed by co-eluent solution. Geraniol and icaridin were quantified by the HPLC method, as described above.

The release profile data for the compounds were analyzed using different mathematical models (zero order, first order, Higuchi, and Korsmeyer-Peppas), in order to investigate the mechanisms of release of the active agents from the different systems.

### *In vitro* Determination of Permeation Profiles

Assays of the permeation kinetics were performed using a two-compartment vertical diffusion cell system (Microette Plus, Hanson Research, Chatsworth, CA, United State) with donor compartment (1.72 cm^2^ permeation area) and receptor compartment (7 ml) separated by a synthetic membrane (Strat-M^®^, 25 mm diameter, Millipore Co., United State) (Uchida et al., 2015; Kaur et al., 2018).

Samples of the liquid formulations (0.6 ml) or hydrogels (0.6 g) were placed in the donor compartment, in contact with the membrane, and the receptor compartment was filled with 0.1 M sodium phosphate buffer (pH 7.4). The system was kept under magnetic stirring (350 rpm), at 32.5 ± 0.5°C. Aliquots were removed at times of 0, 0.5, 1, 2, 4, 6, 8, 12, and 24 h, for analysis using HPLC. All the formulations were evaluated in triplicate. The cumulative amount of repellent permeated, Q (μg/cm^2^), that permeated through the Strat-M per unit area was plotted as a function of time (min). For data analysis, the flux values were obtained from the slope of the concentration versus time curve in the linear portion of plot (interval between 15 and 450 min). The data were analyzed according to Equation 1.

J=P x Cd (1)

where, J (%.cm^−2^. min^−1^) is the flux of the compound through the membrane, P (cm.min^−1^) is the permeability coefficient, and Cd (%.cm^−3^) is the concentration of the compound in the donor compartment (de Araujo et al., 2010). Lag time (t_lag_) was calculated from the intercept (Y = 0).

### Accelerated Stability Assays and Microbiological Control of the Nanostructured Lipid Carriers in Gel

The accelerated stability study of the NLC formulations in gel was performed according to the Harmonized Tripartite Guideline for Stability Testing of New Drug Substances and Products Q1A (R2) established by the International Conference on Harmonization, Geneva, Switzerland (European Medicines Agency, 2018) at the following conditions: 40 ± 2°C and 75 ± 5% relative humidity for 6 months. The parameters analyzed during this period were color, odor, density, pH, and viscosity (ISO 6658, 2005). The color and odor were evaluated by observation of macroscopic changes. The hydrogen ion potential (pH) was determined using an electrode immersed in an aqueous dispersion containing 10% of the sample. The specific density was determined from the mass and volume of the sample. In addition, the stabilities of the formulations were periodically evaluated by oscillatory frequency analysis, considering the elastic (G’) and viscous (G”) moduli. The formulations were stored in tubes (80% LDPE +20% HDPE), under controlled conditions of temperature (40 ± 2°C) and relative humidity (75 ± 5% RH).

Microbiological control of non-sterile cosmetic formulations requires determination of the total number of microorganisms present (ISO 16212, 2017; ISO 21149, 2017), as well as identification of pathogens such as *Salmonella* sp., *Escherichia coli*, *Staphylococcus* sp. (ISO 22718, 2015), and *Pseudomonas aeruginosa* (ISO 22717, 2015), which should not be present (Dao et al., 2018; Sharma Bora et al., 2019). Analyses performed on the day of preparation and after 6 months.

### Statistical Analysis

The data were presented as mean ± standard deviation (SD). The Shapiro-Wilk test was used for evaluation of normality, followed by application of the two-way ANOVA test for multiple variables, considering a significance level of 0.05. The software used was GraphPad Prism 8.0.1.

## Results and Discussion

### Characterization and Stability of the Nanostructured Lipid Carriers in Suspension

**FIGURE 1:**
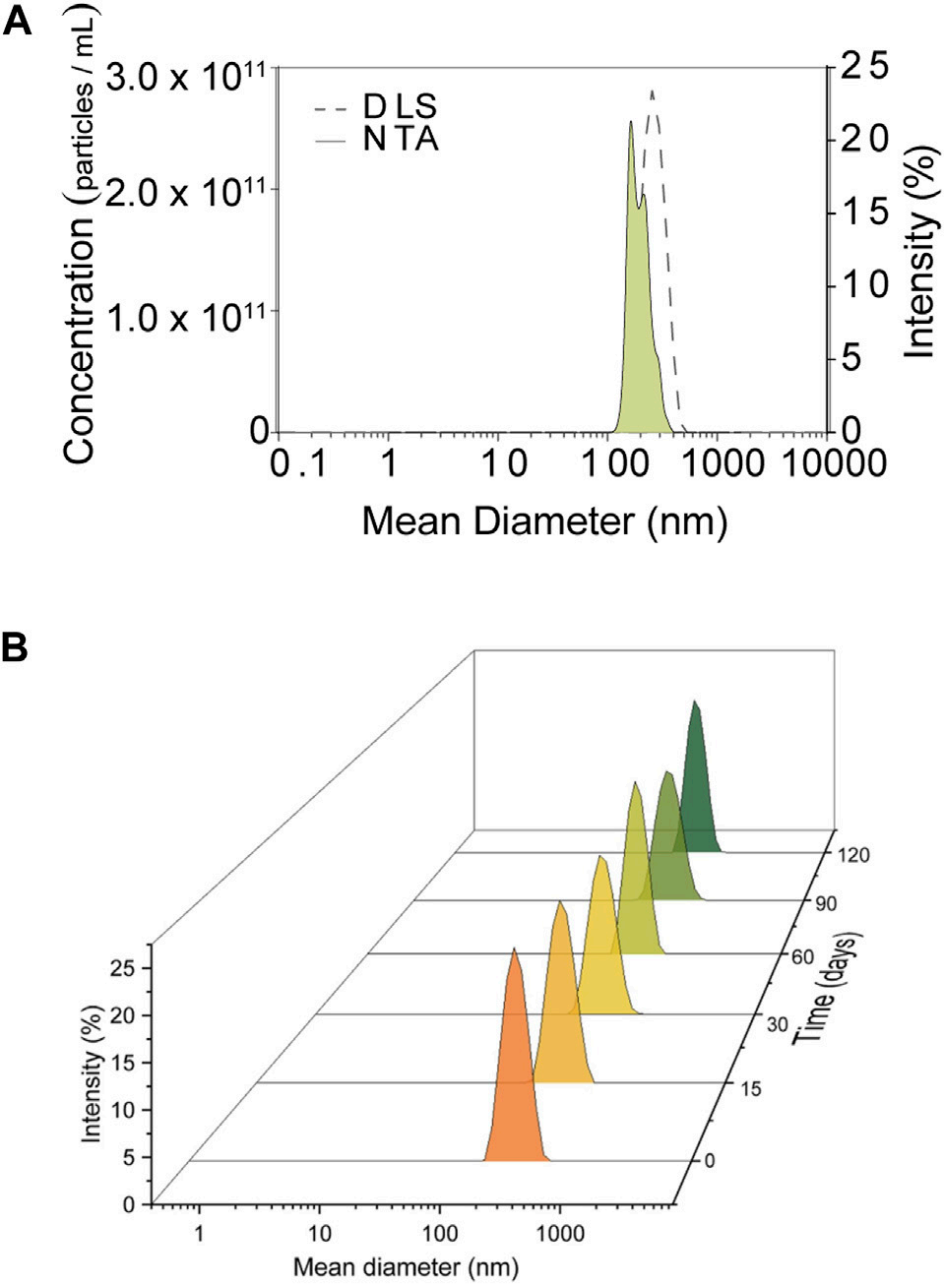
Size distribution profile and concentration of the NLCs over time (at 0, 15, 30, 60, 90, and 120 days), obtained by DLS and NTA, for the suspension of NLCs loaded with geraniol (GRL) and icaridin (ICA). The analyses were performed in triplicate (n = 3).

The results of the physicochemical characterizations of the systems are provided in Table 1 and Figure 1. The control formulation (NLC) presented an average diameter of 271 ± 5 nm, while the value for the repellent formulation (NLC_ICA + GRL) was 252 ± 5 nm, both with low polydispersity index (<0.1). Use of the DLS and NTA techniques resulted in different mean sizes of the nanoparticles loaded with repellents, although similar size distributions were obtained using the two techniques, with low polydispersity of 0.46 ± 0.07, in agreement with the polydispersity calculated from the DLS measurements (Figure 1A). The size distribution profile analyzed for 120 days showed good stability of the system, with no significant differences (*p* < 0.05) in formulation homogeneity (Figure 1B). The negative ZP values were due to the type of surfactant used (PVA), with the stabilization mechanism being according to steric hindrance, rather than electrostatic effects, which commonly leads to lower ZP values. In a stability study of PLGA nanoparticles loaded with insulin, high molecular weight PVA had a greater stabilizing effect than Tween 20:Tween 80 or low molecular weight PVA (Rawat et al., 2015).

**TABLE 1:** Characterization of the NLCs without repellents and containing geraniol (GRL) and icaridin (ICA): Dynamic light scattering (DLS) analyses of mean diameter (MD) and polydispersity index (PDI); nanotracking analysis (NTA) of mean diameter (MD) and NLCs concentration (CT); microelectrophoresis analysis of zeta potential (ZP); and ultracentrifugation/HPLC analysis of encapsulation efficiency (EE). The analyses were performed in triplicate (mean ± SD).

Formulations	MD		PDI	ZP (mV)	CT (x10^13^particles/mL)	EE (%)
	DLS	NTA				
NLC	272 ± 5	211.3 ± 2	0.064 ± 0.04	-17 ± 1.2	2.0 ± 0.08	—
NLC_ICA + GRL	252 ± 5	209.4 ± 1	0.065 ± 0.02	-14 ± 0.2	2.5 ± 0.1	ICA 99.4
	—	—	—	—	—	GRL 99.9

The results obtained for reproducibility and repeatability over 60 days are shown in Supplementary Figure S1 (Supplementary Material). The stability profile over 60 days revealed insignificant (*p* < 0.05) changes of the mean particle diameter and polydispersity index, indicating a monodisperse formulation and high stability of the system for the five batches analyzed. During the same period, the EE for icaridin ranged between 99.4 ± 0.3% and 87 ± 0.1%, while the EE for geraniol ranged between 99.9 ± 0.04% and 97 ± 0.06%. The NLC characterization results agreed with some previous studies. For example, Song et al. (2016) investigated the effect of the liquid/solid lipid ratio on NLCs loaded with furbiprofen, which had average size <200 nm, zeta potential < −20 mV, and encapsulation efficiency >78%. No changes in these parameters were observed after 90 days of storage at temperatures of 4 and 25°C.

Pinto et al. (2014) investigated NLCs loaded with methotrexane, for topical use in the treatment of psoriasis. In agreement with the present work, the average particle size was 274–298 nm, with PDI <0.25. The mean pH of the formulations was 6.2 ± 0.3, with no significant changes observed over time. Similarly, Motawea et al. (2018) developed an NLCs formulation for topical use, obtaining pH values between 5.6 and 6.4, which were within the permissible range.

### Morphologies of the Nanostructured Lipid Carriers in Suspension and Gel

**FIGURE 2:**
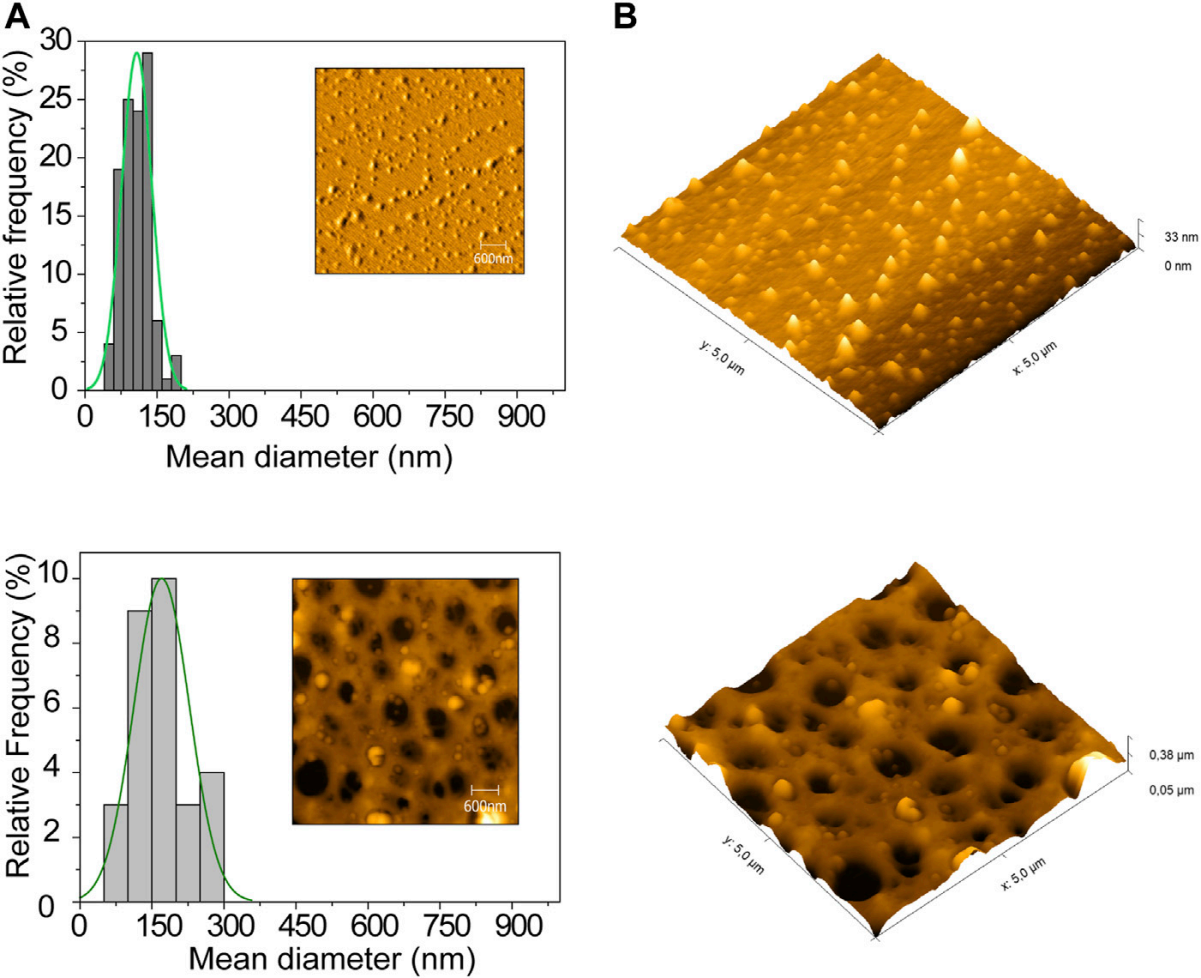
Atomic force microscopy results (area of 5 μm × 5 μm): **(A)** Size distribution profile and surface topography image of the NLCs loaded with the active agents, in suspension and entrapped in gel; **(B)** 3D topography of the NLCs in suspension and entrapped in gel.

In addition to the DLS and NTA analyses, atomic force microscopy was used to evaluate the size distributions and morphologies of the nanoparticles. The nanoparticles presented mean diameters between 90 and 150 nm, as shown in Figure 2A (analysis of 100 particles using Gwyddion software). In this case, the size was smaller than obtained using DLS and NTA, due to the specific characteristics of the different techniques. For analysis using AFM, the sample must be dried, which can lead to changes in the morphology, especially regarding particle organization and distribution, depending on the concentration and osmolarity of the particles (Sitterberg et al., 2010). On the other hand, the low polydispersity index was in agreement with the DLS and NTA results. Figure 2B shows the 3D topography of the NLCs in gel (HPMC), confirming that the NLCs were inserted in the polymeric gel network.

It should be noted that the mean size of the NLCs incorporated in the gel was compatible with that of the NLCs in suspension. Therefore, the incorporation process did not lead to alteration of the NLC structure, size, or morphology, and there was no effect on particle aggregation, showing that the NLCs remained stable after incorporation in the hydrogel. There have been various reports of AFM used to analyze the topography of hydrogels (Helfricht et al., 2017). El-Mekawy and Jassas (2017) described the synthesis of insulin systems employing ZnO nanoparticles in chitosan hydrogel, with analyses using AFM, transmission electron microscopy (TEM), and scanning electron microscopy (SEM), showing that the film exhibited porosity and that nanoparticles were present in the matrix, corroborating the present findings.

### Differential Scanning Calorimetry Analyses

The DSC thermograms were used to investigate the interactions of the various formulation components, as well as the effect of the preparation process on the components. The thermogram obtained for tripalmitin, the main component of the NLC lipid matrix, showed an endothermic peak at around 66°C (Supplementary Figure S4 I, Supplementary Material). The other thermograms corresponding to the components of the control NLCs (Supplementary Figure S4 II), the NLCs in suspension (Supplementary Figure S4 III), and the NLCs in gel (Supplementary Figure S4 V) showed a shift of the phase transition temperature, due to interaction between the charges of the polymeric components and the formation of nanoparticles. In addition, the presence of the active agents (icaridin and geraniol) led to a reduction of the endothermic peak temperature from 61°C (without the active agents) to 56°C (with icaridin and geraniol) (Supplementary Table S1). The thermogram for HPMC showed a broad endothermic signal in the temperature range between 60 and 130°C, which could be attributed to the water loss (Supplementary Figure S4 IV). However, this peak did not appear in the thermogram for the formulation of NLCs incorporated in the HPMC gel, which could have been related to the complete dispersion of the NLCs in the polymeric matrix of the gel (McPhillips et al., 1999; Jayaramudu et al., 2021).

According to the literature, this behavior could be indicative of changes in the structural organization of the lipid crystalline network following interaction with the active agents, leading to a reduction of the tripalmitin melting point (Sarangi et al., 2018). Furthermore, the mixing of two lipids usually leads to a lower melting temperature and lower enthalpy, due to structural disruption of the solid lipid (Doktorovova and Souto, 2009). A similar finding was reported by Kudarha et al. (2015), who analyzed the structure of NLCs with encapsulated bicalutamide (a drug used to treat prostate cancer) and observed a slight change in the melting point of the lipid matrix after interaction with the drug.

In another study evaluating the nanoencapsulation of geraniol in chitosan/gum arabic nanoparticles, it was also observed that the presence of geraniol decreased the nanoparticle endothermic peak, probably due to interaction of the matrix with the hydrophobic encapsulated component (de Oliveira et al., 2018). Teeranachaideekul et al. (2007) used DSC to investigate the interaction of ascorbyl palmitate with the NLC lipid matrix, observing a shift of the maximum peak (56.9°C), because the active agent altered the crystallization process of the NLC lipid matrix.

### Rheological Analysis

Rheological analysis of the formulations provided information concerning the effects of composition and storage time on the mechanical properties of the hydrogels before and after incorporation of the NLCs. For this, flow and oscillatory analyses were used to obtain parameters including the Table 2 elastic modulus (G’), viscous modulus (G”), and apparent viscosity (η*).

**TABLE 2:** Rheological parameters G’ (elastic modulus), G” (viscous modulus), and η (viscosity), measured at a frequency of 1 Hz for the control hydrogel (HPMC), the emulsion with 2% GRL and 5% ICA, the NLCs formulation (hydrogel) without active ingredients, and the NCLs formulation (hydrogel) with 2% GRL and 5% ICA.

Parameters		Formulation in HPMC		
	Control	Emulsion_ ICA + GRL	NLC	NLC_ICA + GRL
G’ (Pa)	10.62	17.120	35.49	45.14
G” (Pa)	22.11	33.58	51.5	62.73
G’/G’’	0.480	0.509	0.689	0.719
η (Pa.s) 32.5°C	5,531	5,999	9,954	12,300

**FIGURE 3:**
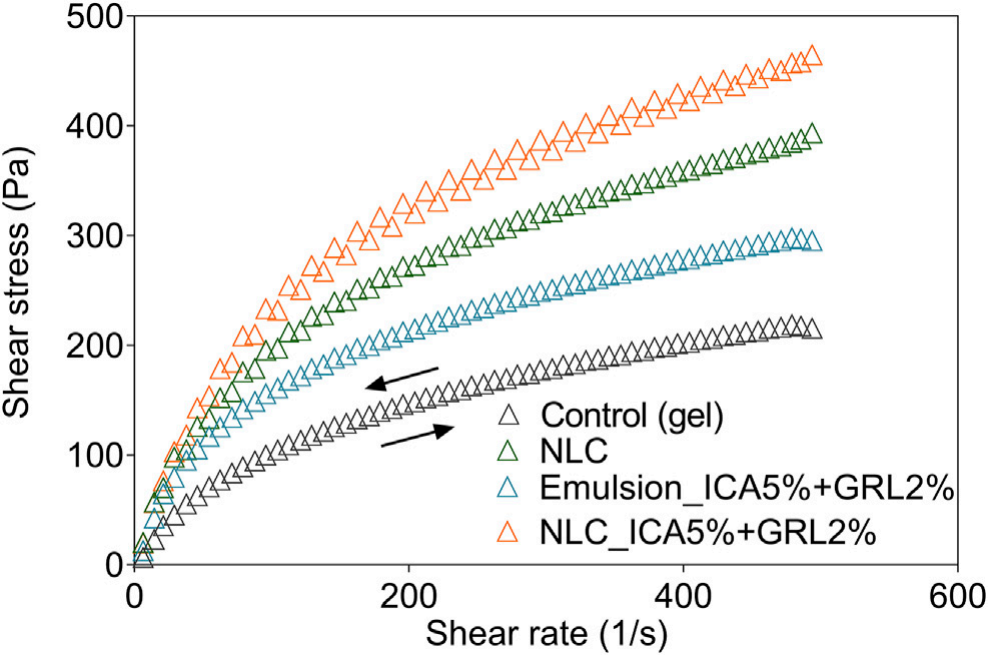
Rheograms of shear stress variation according to shear rate for the control HPMC hydrogel, the NLCs formulation without active ingredients, the emulsion with 2% geraniol (GRL) and 5% icaridin, and the NLCs formulation with 2% geraniol and 5% icaridin.

The flow curve profiles were indicative of pseudoplastic behavior, characteristic of some non-Newtonian fluids, where high shear rates result in low flow resistance of the polymer particles. The graph of the flow curves shows the ascending curves, when the shear rate is increased over time, and the descending curves, when the inverse process occurs. This is known as the hysteresis cycle (Lee et al., 2009). In the present case, the ascending and descending curves were almost superimposed (Figure 3).

The calculated flux index (n) values were all lower than 1, indicating that the formulations presented thixotropic behavior, even after incorporation of the NLCs. Transient and reversible interactions between the NLC surfaces and hydrophilic groups in the HPMC structure may act to modulate the self-organization of hydrogels, so that there is complete recovery of their mechanical properties, even under high applied shear stress, as shown by a small hysteresis area (Appel et al., 2015).

It should be noted that pseudoplastic and thixotropic behaviors are essential for topical formulations, so that they deform during application, becoming more fluid and easy to spread, and then return to their initial structural organization, preventing flow of the product away from the application site (Malkin and Isayev, 2017). Thixotropic fluids are known to decrease the apparent viscosity with the time of application at a given shear rate, which is reversible phenomena. This decrease in viscosity can be explained by the gradual loss of the structure formed by particles in the dispersed phase, whose bond strength does not resist the action of the applied shear. In addition, when the shear force ceases, the fluid returns to its initial structural conformation (Ghica et al., 2016). A thixotropic formulation tends to have greater physicochemical stability, with little separation of the components during storage (Gaspar and Maia Campos, 2003). The oscillatory temperature analysis and the G’ and G” values show that the NLCs incorporated in gel showed greater stability than emulsion in gel. It was possible to observe that a disruption of the gel occurs at temperatures above 35°C (Supplementary Figure S5, Supplementary Material). Corroborating with a study that evaluated the thermo-responsive behavior of HPMC, which verified the abrupt drop in modulus on heating. It also can account for the decreased stability of the fibrils, such that they melt over a range of temperatures (≥40°C) which resulted in a decrease in viscosity that was also possible to verify in our study (Lodge et al., 2018). The oscillatory frequency analysis and the G′ and G″ values showed that the fluidity decreased in the presence of the NLCs (increase of the G’/G” ratio), while the viscosity increased at higher shear rates, due to the dispersion of the particulate phase in the gel polymeric matrix (Table 3; Supplementary Figure S6, Supplementary Material). The values of ŋ, at 1 Hz, were from 5,531 to 12,300 Pa.s, in the order: control < gel emulsion < empty NLCs gel < loaded NLCs gel (Supplementary Table S4). Previous work has described the effects of incorporation of solid lipid nanoparticles in gels to increase the viscosity of topical formulations (Lippacher et al., 2004).

**TABLE 3:** Parameters for permeation of geraniol (GRL) and icaridin (ICA) across Strat-M^®^ membranes from the suspension and gel formulations of NLCs containing 5% ICA +2% GRL.

Formulations	Flux (10^−4^.μg.cm^−2^.min^−1^)	Permeability coefficient (10^−4^.cm.min^−1^)	Lag time (min)
NLC suspension	—	—	—
GRL	1.0	0.5	591.4
ICA	109.1	21.8	23.8
NLC gel	—	—	—
GRL	44.4	22.2	43.9
ICA	1,545	309	24.3

### Release Behaviors of the Nanostructured Lipid Carriers in Suspension and Gel

**FIGURE 4:**
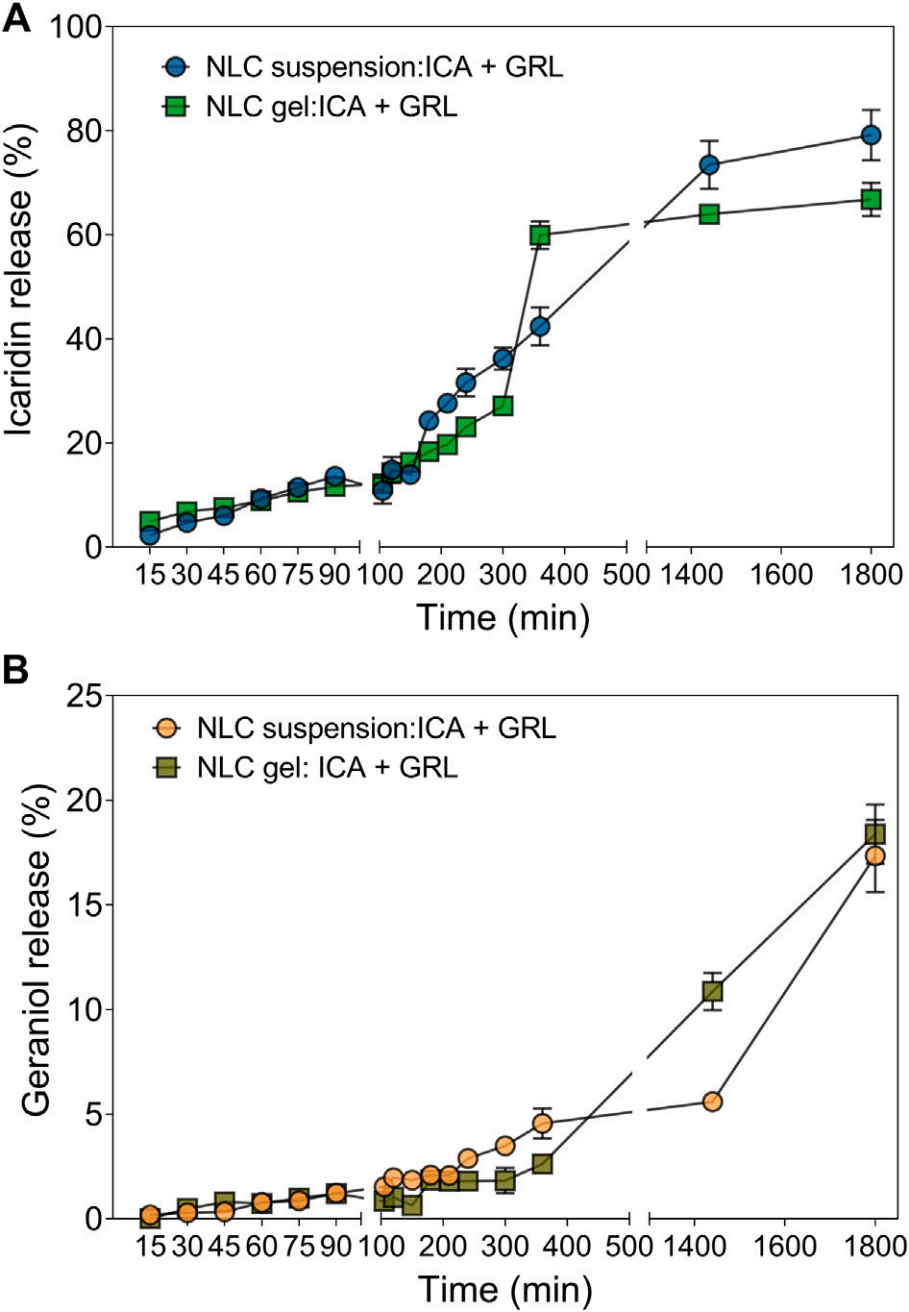
Cumulative release profiles for the repellents in the suspension and gel formulations. The NLCs contained 2% geraniol (GRL) and 5% icaridin (ICA). The system was maintained under magnetic stirring (300 rpm), at 32.5°C, for 32 h. The analyses were performed in triplicate (the values are shown as mean ± SD).

The profiles of release of icaridin and geraniol from the NLCs are shown in Figure 4. For icaridin, the release percentages were 79 ± 5% and 67 ± 3% for the NLCs in the suspension and gel formulations, respectively, after 30 h. For geraniol, the release percentages were 17 ± 2% and 18 ± 1% for the NLCs in the suspension and gel formulations, respectively, after 30 h. This difference between the release profiles of the two compounds could have been due to their different physicochemical characteristics, since the solubility of icaridin in water is 8.2 g/L, at 20°C, while that of geraniol is 0.1 g/L, at 20 °C (PubChem, 2019a; 2019b).

Furthermore, it is important to mention that although the encapsulation of both active ingredients led to a delayed release profile when compared to unencapsulated systems (emulsions) for both suspension and gel formulations this is not a problem from the point of view of the start of repellent activity, since it is a system that co-encapsulates two active ingredients, it is possible to observe that the icaridin that is released more quickly would be responsible for the immediate repellent effect, while the geraniol would be responsible for the longer repellent effect.

The release of icaridin from the NLCs in suspension was significantly higher (*p* < 0.05) than from the NLCs in the gel. This difference in the release rate was probably due to the entrapment of the nanoparticles in the HPMC polymeric matrix, which has a dilation rate (SR) of 96 h, enabling the integrity of the matrix to be maintained for an extended period (Notario-Pérez et al., 2019). A similar effect was observed for a vaginal film for sustained release of the drug tenofovir (TFV), which prevents HIV infection. In this case, the combination of HPMC and zein containing TFV, at a ratio of 1:5, provided sustained release of the drug for 24 h, while addition of 40% PEG increased the release time to 120 h, due to greater plasticity of the matrix (Notario-Pérez et al., 2019).

Comparison was also made between the NLCs and the emulsion containing the repellents, with sustained release profiles observed in both cases (Supplementary Figure S7, Supplementary Material). The profile of sustained release from the emulsion could be explained by the formation of micellar structures during the process of emulsification with the surfactant (PVA). Recent work has described the use of these micellar structures with drugs that have low water solubility, with the aim of reducing adverse effects (Yukuyama et al., 2017).

Mathematical modeling was used to describe the dissolution/release profiles (Caccavo, 2019), in order to obtain a better understanding of the release kinetics of the repellents. Based on the correlation coefficient (r^2^) values, the Korsmeyer-Peppas model provided the best description of the release of icaridin in both liquid and gel formulations (Supplementary Table S5). This model is a derivation of the Higuchi model, but in addition to Fickian release, the release mechanism also includes the contribution from relaxation of the polymeric chains of the gel matrix. The value of n (0.97) obtained for the release of icaridin from the NLCs in the suspension corresponded to non-Fickian release kinetics, probably because the release depended only on diffusion from the NLCs, not involving diffusion through the medium. On the other hand, the value of n (1.48) obtained for icaridin in the gel formulation was indicative of Super-Case II transport, mainly governed by relaxation of the polymeric chains. For geraniol in the NLCs in suspension, the Higuchi model provided the best description of the release, indicating that the release mechanism was predominantly by diffusion. For the release of geraniol present in the gel formulation, the zero-order mathematical model provided the best fit. This model is most suitable when the rate of release of a substance from a hydrophilic matrix such as HPMC is slow.

It could be concluded from application of the mathematical models that the kinetics of icaridin release from both formulations presented a non-Fickian profile, with a higher n value for the gel, probably due to the influence of two release systems, involving the nanoparticles and the hydrogel. In contrast, the release kinetics of geraniol followed Fick’s law, although incorporation in the gel led to slower diffusion, according to the zero order model. de Oliveira et al., 2019 evaluated the release mechanisms of botanical compounds encapsulated in zein, where the Korsmeyer-Peppas model provided the best representation of the release profiles. For zein nanoparticles containing geraniol and eugenol, n < 0.45 indicated that diffusion was the main release mechanism, while zein nanoparticles containing geraniol and cinnamaldehyde showed an anomalous release profile (a combination of diffusion and case II transport). Another mechanism of geraniol release was studied in the development of a portable device for external use, loaded with geraniol (Kwan et al., 2019). In this case, the zero-order mathematical model was most representative, due to the slow release of geraniol, as also observed in the present work.

Notario-Pérez et al. (2019) reported that for the combination of HPMC and zein with tenofovir, the best description of the profile of release from films was provided by the Higuchi and Hopfenberg model, indicating the importance of diffusion of the drug and erosion of the polymer. Variation of the value of n was observed, where the highest values were attributed to structural alteration of the polymer matrix, while lower values indicated the predominance of diffusion of the drug, in agreement with the present findings.

### *In vitro* Permeation Profiles

Comparison was made of the permeation profiles of the emulsions (5% ICA +2% GRL) and the NLCs (5% ICA +2% GRL) in suspension and gel. There have been many studies concerning the optimization of transdermal permeation systems employing formulations in the form of sprays, gels, and creams. In the case of a repellent formulation, good performance is indicated by prolonged release, low doses, low frequency of applications, and low permeation rates.

**FIGURE 5:**
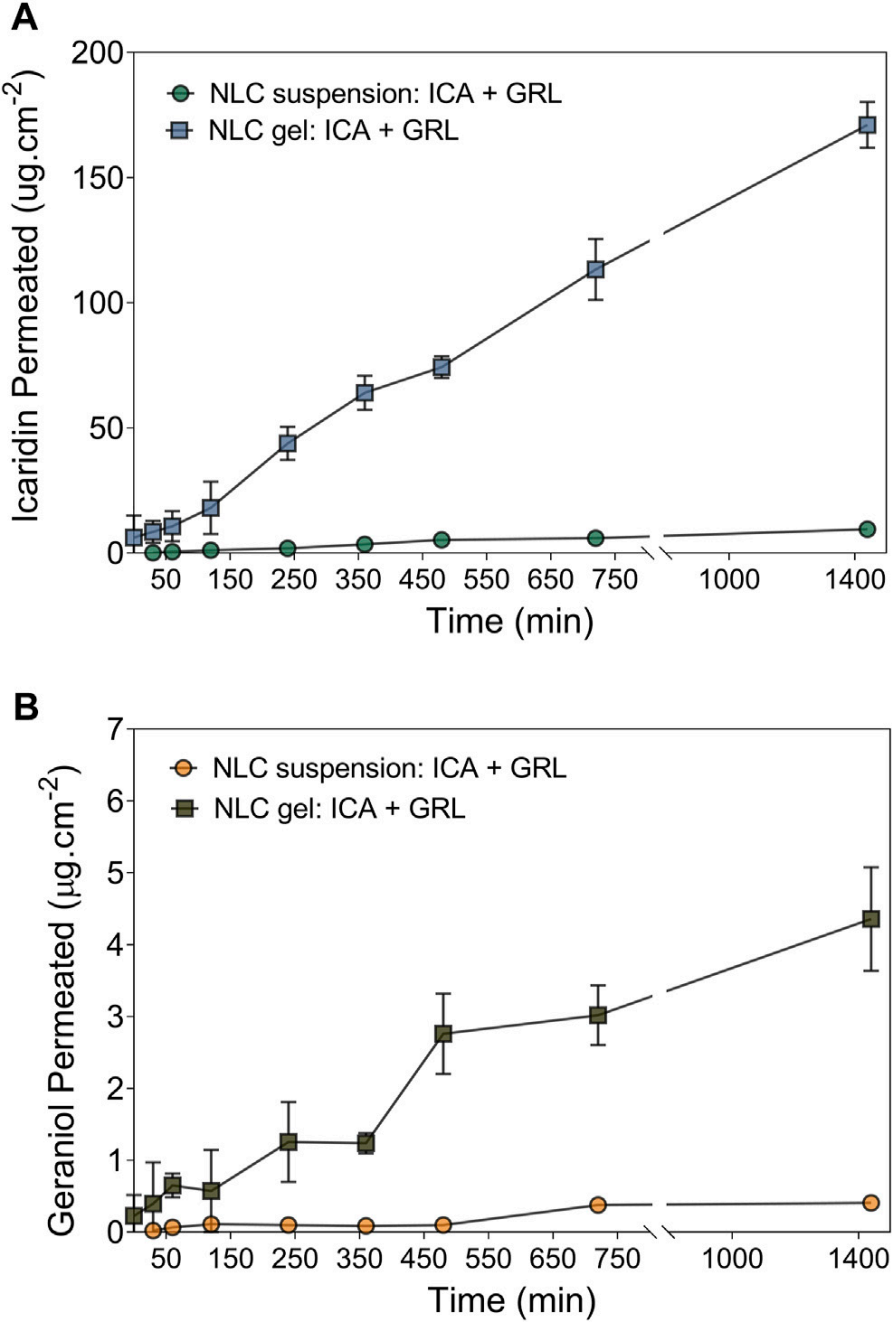
Permeation profiles of the repellents across Strat-M^®^ membranes (mean ± SD, n = 3 per formulation), obtained using a vertical Franz diffusion cell apparatus with the formulations of NLCs containing 5% ICA +2% GRL. **(A)** Icaridin permeated in suspension and gel. **(B)** Geraniol Permeated in suspension and gel.

The Strat-M^®^ synthetic membrane system has been used in cutaneous permeation experiments, due to its easy availability, reproducible results, and similarity to human skin (Uchida et al., 2015; Grillo et al., 2019). Here, the permeation of the repellents was evaluated using the NLCs formulations in suspension and gel. The results are summarized in Figure 5 and Table 3. All the formulations presented gradual permeation during the experiment (24 h). There was a significant difference (*p* < 0.05) between the permeation (during 24 h) of icaridin from the NLCs in suspension (9.5 ± 0.1 μg/cm^2^) and from the gel formulation (171 ± 9 μg/cm^2^). A possible explanation was greater membrane hydration of the NLCs in the hydrogel, which acted to increase penetration of the compound. The degree of porosity and gel hydration can influence the solubility of active compounds in water, leading to rapid release and reduced bioavailability (Doktorovova and Souto, 2009; Desfrançois et al., 2018; Iikura et al., 2019).

Furthermore, HPMC provides film formation increasing the surface area available for permeation (Aho et al., 2017).

The lag time of icaridin to be similar in both formulations. On the other hand, the lag time of geraniol in NLC suspension (591.4 min) was higher than NLC gel (44.4 min). The fluxes and permeability coefficients obtained for icaridin showed significant differences between the formulations in suspension (109.1 × 10^−4^ μg cm^−2^ min^−1^ and 21.8 × 10^−4^ cm^−2^ min^−1^) and gel (1,545 × 10^−4^ μg cm^−2^ min^−1^ and 309 × 10^−4^ cm^−2^ min^−1^). Similarly, there were significant differences for the permeation of geraniol in suspension (1.0 × 10^−4^ μg.cm^−2^ min^−1^ and 0.5 × 10^−4^ cm^−2^ min^−1^) and gel (44.4 × 10^−4^ μg cm^−2^ min^−1^ and 22.2 × 10^−4^ cm^−2^ min^−1^), although the values were lower, compared to icaridin. These results were consistent with the release profiles and could be explained by the fact that the water solubility of icaridin (8.2 mg/ml) is 80 times higher than that of geraniol (0.1 mg/ml) (PubChem, 2019b; 2019a).

The action of a repellent mainly occurs by its volatilization. Therefore, since the vapor pressure of icaridin (4 × 10^−4^ mm Hg) is around 100-fold lower than that of geraniol (3 × 10^−2^ mm Hg), the vaporization of icaridin present in the NLCs gel formulation could proceed in a more sustained manner.

Although some studies have shown that NLCs can enhance the permeation of drugs through the skin (Junyaprasert et al., 2009; Na et al., 2020), the rates of release and permeation depend on the physicochemical properties of the compounds, the polarity of the solvents, the size of the nanoparticles, and the matrices within which they are dispersed. In the present work, low percentages of the compounds permeated through the Strat-M membrane, showing that these repellent formulations could potentially reduce the amounts of active agents permeated through the epidermal barrier, consequently reducing their toxicity. Wissing and Müller (2001) found that a topical formulation of SLNs loaded with tocopherol acetate formed a film containing the active agent, which reduced skin penetration. In other work by the same researchers, the penetration of oxybenzone was evaluated *in vitro* (using a Franz cell) and *in vivo* (by tape stripping), which showed that the permeation rate was related to the concentration of the active agent in the formulation and decreased by 30–60% when SLNs were used. Similar considerations could apply to the systems developed here, where the retention of the NLCs in the HPMC matrix resulted in modulation of permeation of the compounds, so they would be retained in the uppermost layers of the skin, providing a prolonged repellent effect.

### Accelerated Stability Study and Microbiological Control of Nanostructured Lipid Carriers in Gel

**FIGURE 6:**
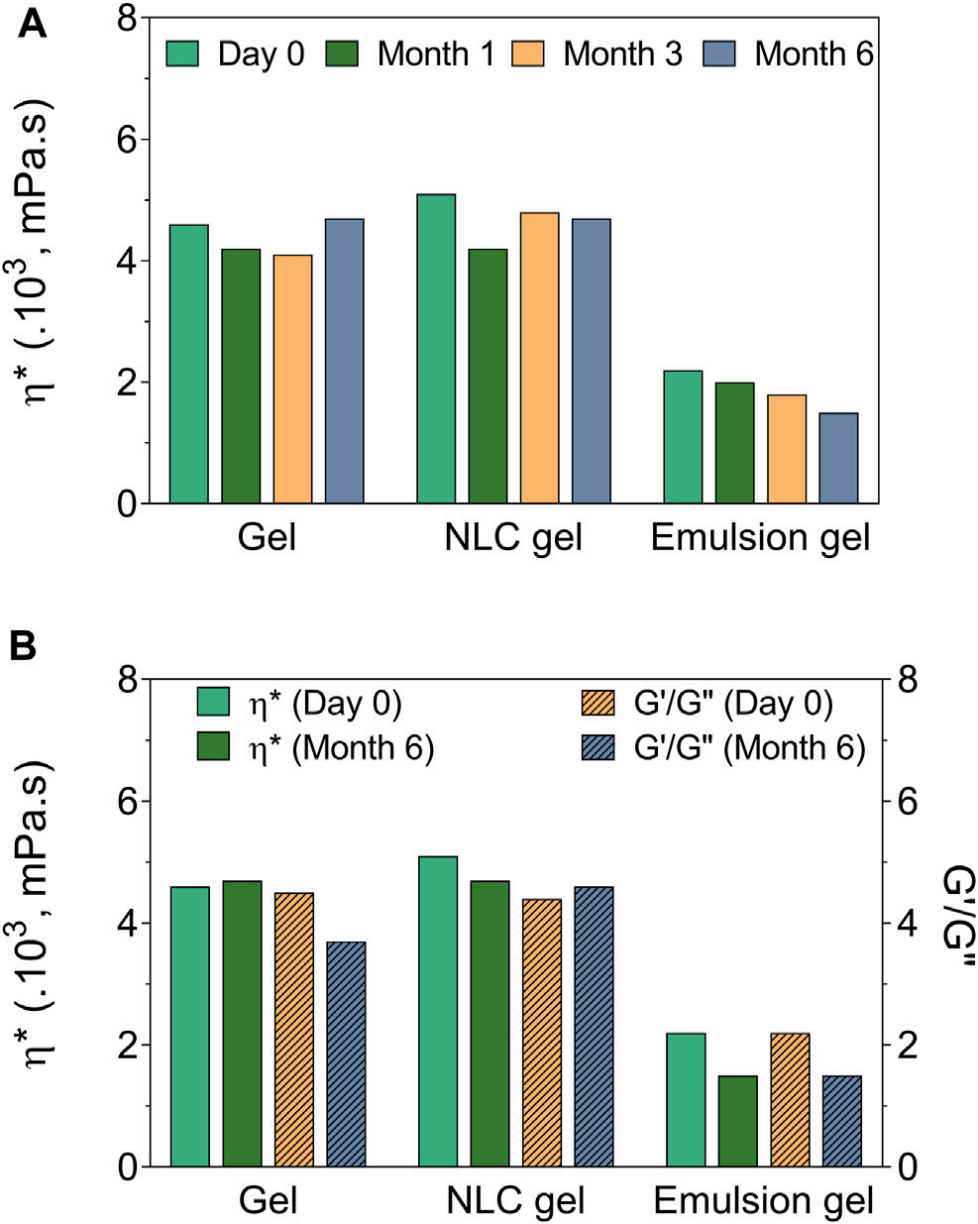
Viscosity and mechanical properties, measured over 6 months, for the gel (2% HPMC), NLCs + gel (2% GRL +5% ICA), and emulsion + gel (2% GRL +5% ICA). **(A)** Apparent viscosity, ​​as a function of time. **(B)** Apparent viscosity (ŋ^*^) and G’/G″ ratio on day 0 and after 6 months.

Evaluation was made of the mechanical properties and viscosity of the formulations, according to storage time, in order to correlate the results with those obtained previously. Figure 6 shows the temporal variation of the apparent viscosity for the formulations, revealing that for the NLCs incorporated in hydrogel, there was no significant alteration of this parameter after 6 months of storage. In contrast, the emulsion incorporated in hydrogel showed a progressive decrease of viscosity, indicating lower physicochemical stability of this system, compared to the NLCs in gel (Figure 6A). The G’/G″ ratios indicated a predominance of the elastic modulus (G’), relative to the viscous modulus (G”), after preparation and up to 6 months of storage, demonstrating the superior maintenance of the structural organization of the NLCs formulation, compared to the emulsion hydrogel (Figure 6B). The organoleptic characteristics of the NLCs formulation, such as color, odor, appearance, and homogeneity, did not change significantly during 6 months, as indicated in Supplementary Figure S8 (green arrow). On the other hand, the emulsion presented phase separation, in agreement with the viscosity analysis and indicating lower physicochemical stability (Supplementary Figure S6, white arrow).

All the formulations presented a significant (*p* < 0.05) decrease of the pH after 60 days (Supplementary Table S3). This could be explained by increased solubilization of the repellent compounds, or by interactions among the formulation components. It is also possible that the formulations could have been degraded by hydrolysis, which indicated the need for microbiological evaluation, for the purpose of quality control. It is known that the skin has a slightly acidic pH, which provides bactericidal and fungicidal activity on the surface. Therefore, it could be concluded that the NLCs and emulsion formulations complied with established safety standards (pH 4.00–7.00) (Hashem et al., 2011; Sharma Bora et al., 2019).

The densities of the formulations were evaluated during the same period (Supplementary Table S4), revealing significant increases (*p* < 0.05). This could have been due to the loss of water, consequently decreasing the volume. The stability was not affected by the incorporation of air (which would increase the volume), considering the increases in density from 1.04 to 1.11 g/cm^3^ and from 0.99 to 1.11 g/cm^3^ for the NLCs and emulsion formulations, respectively. Although stability guidelines do not provide a default density value, a typical value is around 1.003 g/cm^3^. Sensorial analysis showed that there were no significant changes in the organoleptic characteristics of the NLCs formulation (considering color, odor, appearance, and homogeneity). As noted by Isaac et al. (2008), the homogeneity and color of cosmetic products are important aspects from the commercial perspective, since they can influence the decision of the consumer at the time of purchase.

The assays of microbial growth showed an absence of aerobic bacteria and the presence of fungi and yeasts (<1 CFU/g) at levels within the limits established for non-sterile products (Supplementary Table 5S). The results for microbial counts and pathogens showed compliance with quality assurance specifications. Therefore, the increase of pH in the formulations was not associated with contamination.

## Conclusion

A system was developed for simultaneous loading with two repellent compounds, based on the incorporation of NLCs in a polymeric hydrogel. Analyses were made of the compositions, characteristics, and physicochemical stabilities of formulations containing NLCs loaded with geraniol and icaridin, in suspension and incorporated in a hydrogel. The stability profile obtained during 120 days for the NLCs formulation revealed that there were no significant (*p* > 0.05) changes of the mean particle diameter or polydispersity index, indicating high colloidal stability of the system. The high efficiency of encapsulation (>99%) of the compounds in the NLCs resulted in a prolonged release system, with the synthetic and natural repellents being released at different rates, favoring possible synergistic action and an increased spectrum of repellent activity. The formulations presented suitable rheological characteristics, with pseudoplastic and thixotropic behavior that ensured easy spreading, as well as satisfactory physicochemical stability. The *in vitro* permeation profiles showed gradual penetration of the compounds, but with low flux and permeation coefficient values for both geraniol and icaridin, suggesting that the rate of penetration through the epidermal barrier would be low.

## Data Availability

The raw data supporting the conclusions of this article will be made available by the authors, without undue reservation.
