# Supplementary material for: Development of a Mosquito Repellent Formulation Based on Nanostructured Lipid Carriers
Source: Front Pharmacol. 2021 Oct 11;12:760682. doi: 10.3389/fphar.2021.760682 (PMC8542870; doi:10.3389/fphar.2021.760682)

**Supplementary Material**
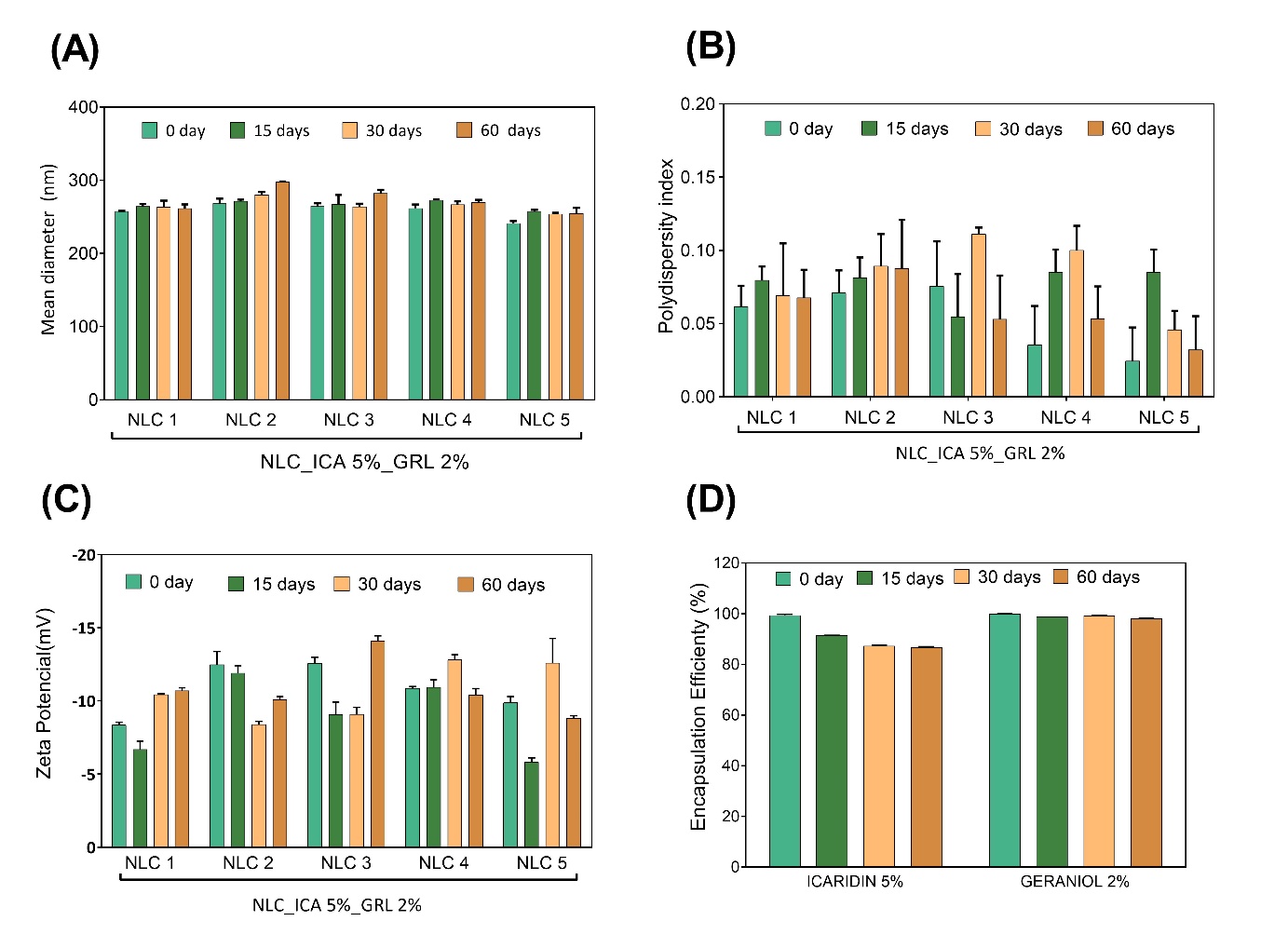


**Supplementary Figure 1.** Properties of the NLCs according to time (at 0, 15, 30, and 60 days): (A) size, (B) polydispersity index, (C) zeta potential, and (D) encapsulation efficiency. Values ​​obtained as mean ± SD (n = 3) for 5 batches of identical NLCs formulations loaded with 5% icaridin (ICA) and 2% geraniol (GRL).


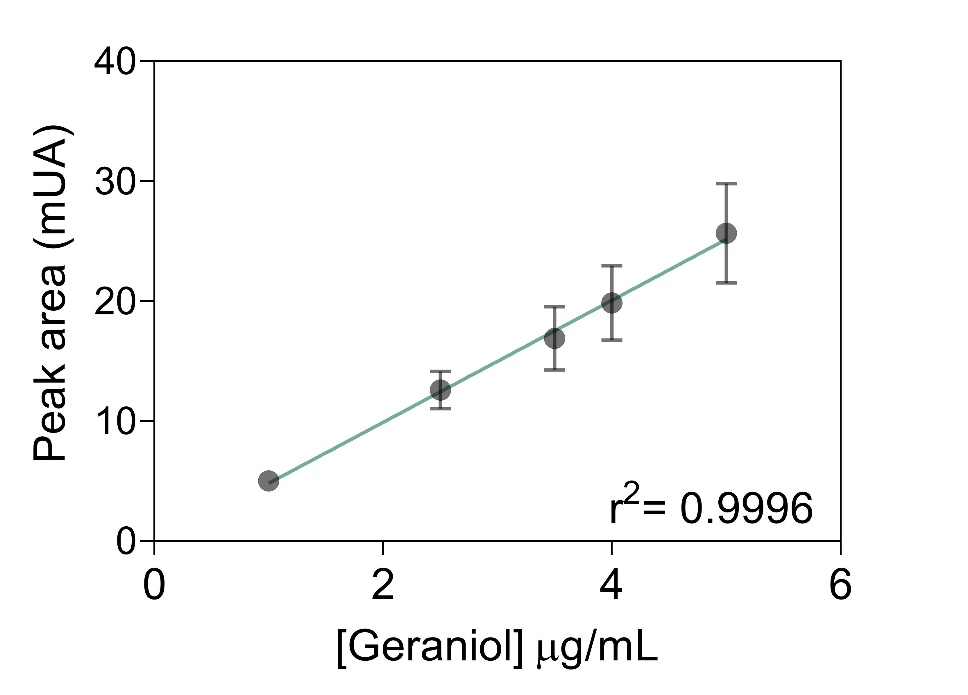


**Supplementary Figure 2**. Analytical curve for geraniol at concentrations between 1 and 5 μg/mL. The values ​​correspond to the mean ± SD (n = 9) of 3 different curves. The analyses were performed using a Phenomenex Gemini C18 column (150 x 4.60 mm, 5 µm), at 25 °C, with 40:60 v/v water:acetonitrile mobile phase at a flow rate of 1 mL/min.


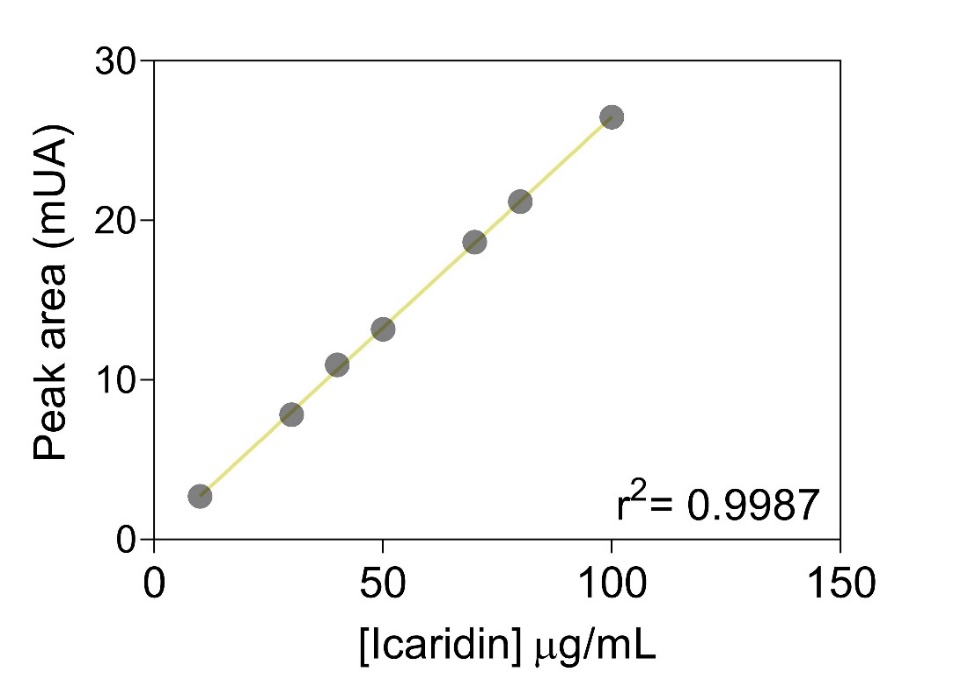


**Supplementary Figure 3.** Analytical curve for icaridin at concentrations between 10 and 100 μg/mL. The values ​​correspond to the mean ± SD (n = 9) of 3 different curves. The analyses were performed using a Phenomenex Gemini C18 column (150 x 4.60 mm, 5 µm), at 25 °C, with 35:65 v/v water:methanol mobile phase at a flow rate of 1 mL/min.


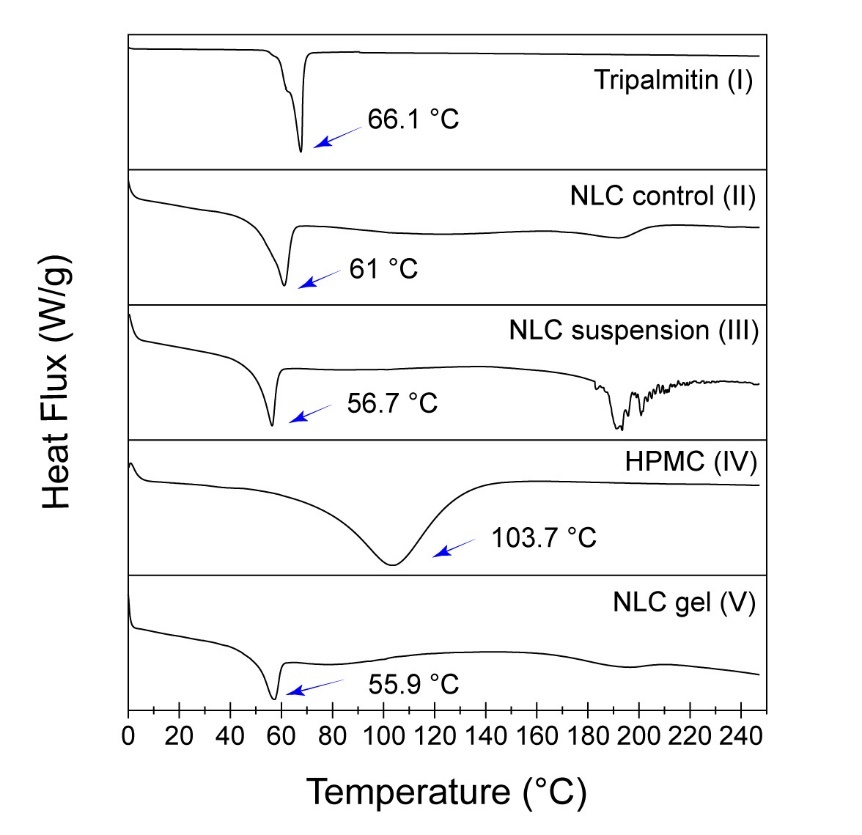


**Supplementary Figure 4.** Differential scanning calorimetry (DSC) thermograms of (I) the NLC main matrix (tripalmitin), (II) the NLCs control (without active agents), (III) the suspension of NCLs loaded with 2% GRL + 5% ICA, (IV) the hydroxypropylmethylcellulose gel matrix, and (V) the NLCs loaded with 2% GRL + 5% ICA and incorporated in gel. The system was heated from 10°C to 250 °C, at a rate of 10 °C/min. Arrows indicate T peak (°C).


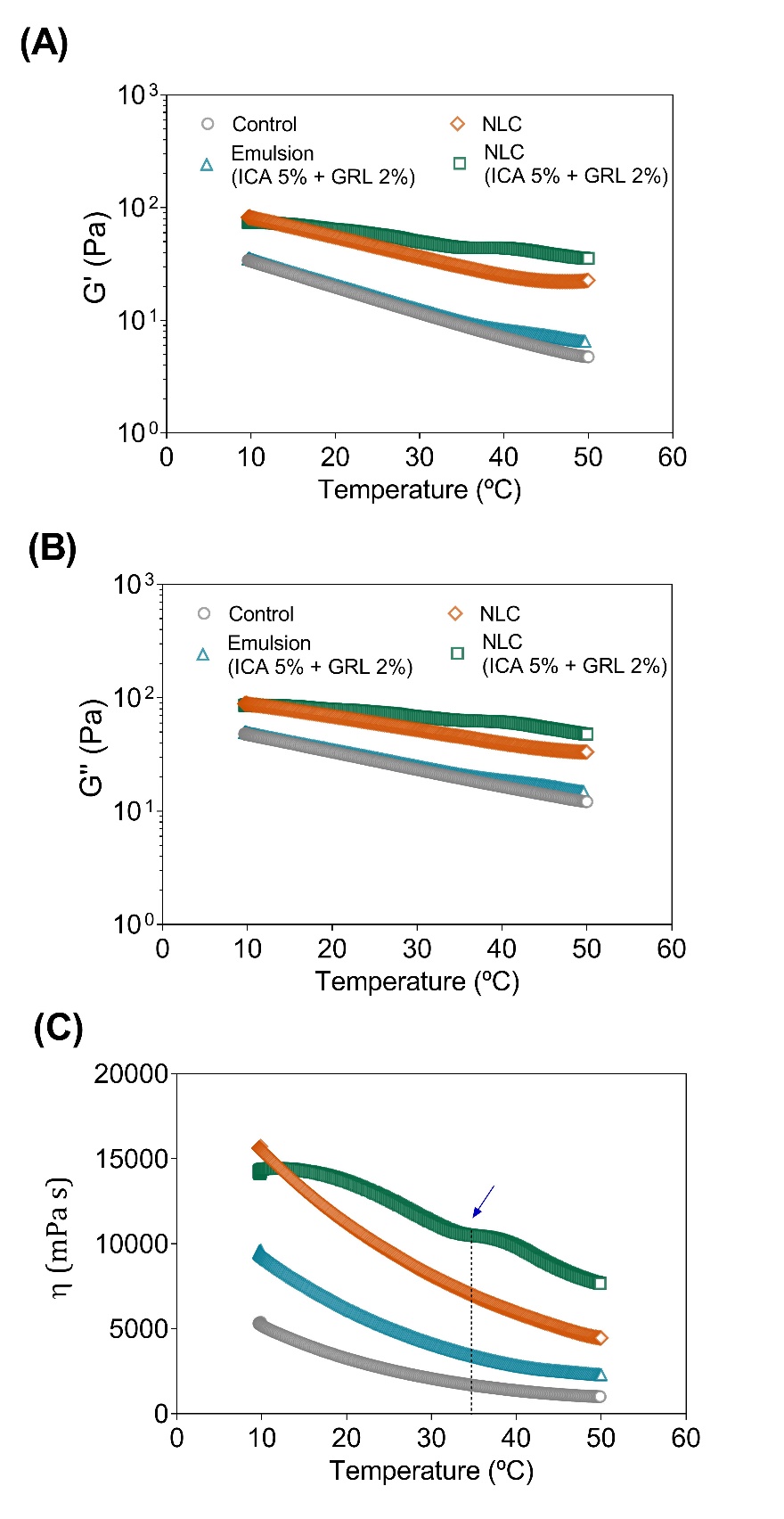


**Supplementary Figure 5.** Storage (A), loss modulus (B), and viscosity (C) at the indicated formulations upon heating; 5 °C/min, 1Hz, from 10°C to 50°C. Control (HPMC); NLC formulation without active ingredients; Emulsion with 2% geraniol (GRL) and 5% icaridin, and the NLCs formulation with 2% geraniol and 5% icaridin. Arrows indicate drop in viscosity (disruption of gel) on heating in NLC.


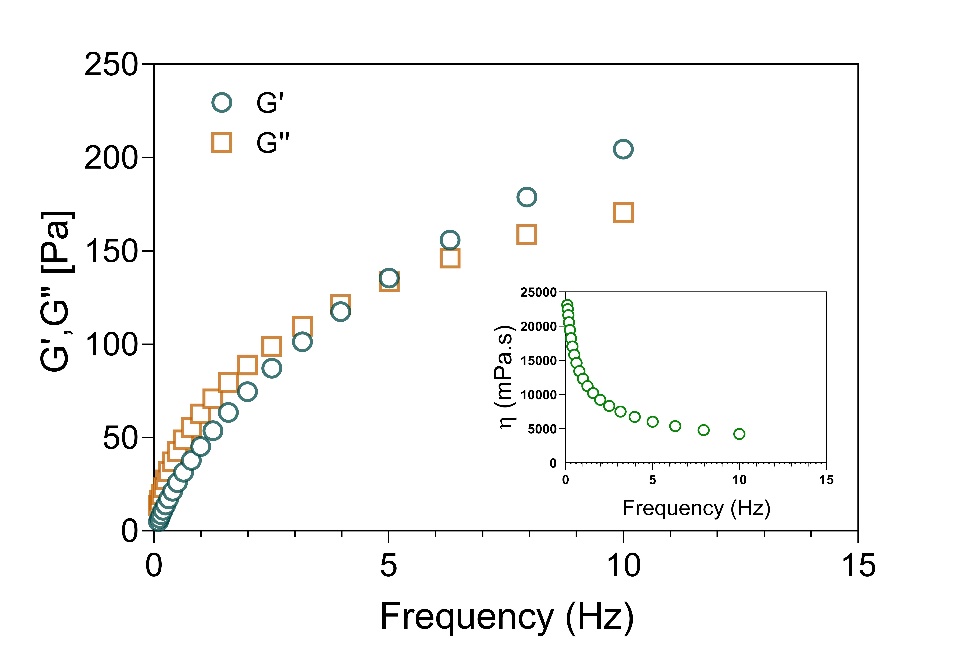


**Supplementary Figure 6.** Rheological analysis of NLC formulation loaded with ICA 5% and GRL 2%. Storage, loss modulus, and viscosity upon frequency variance.


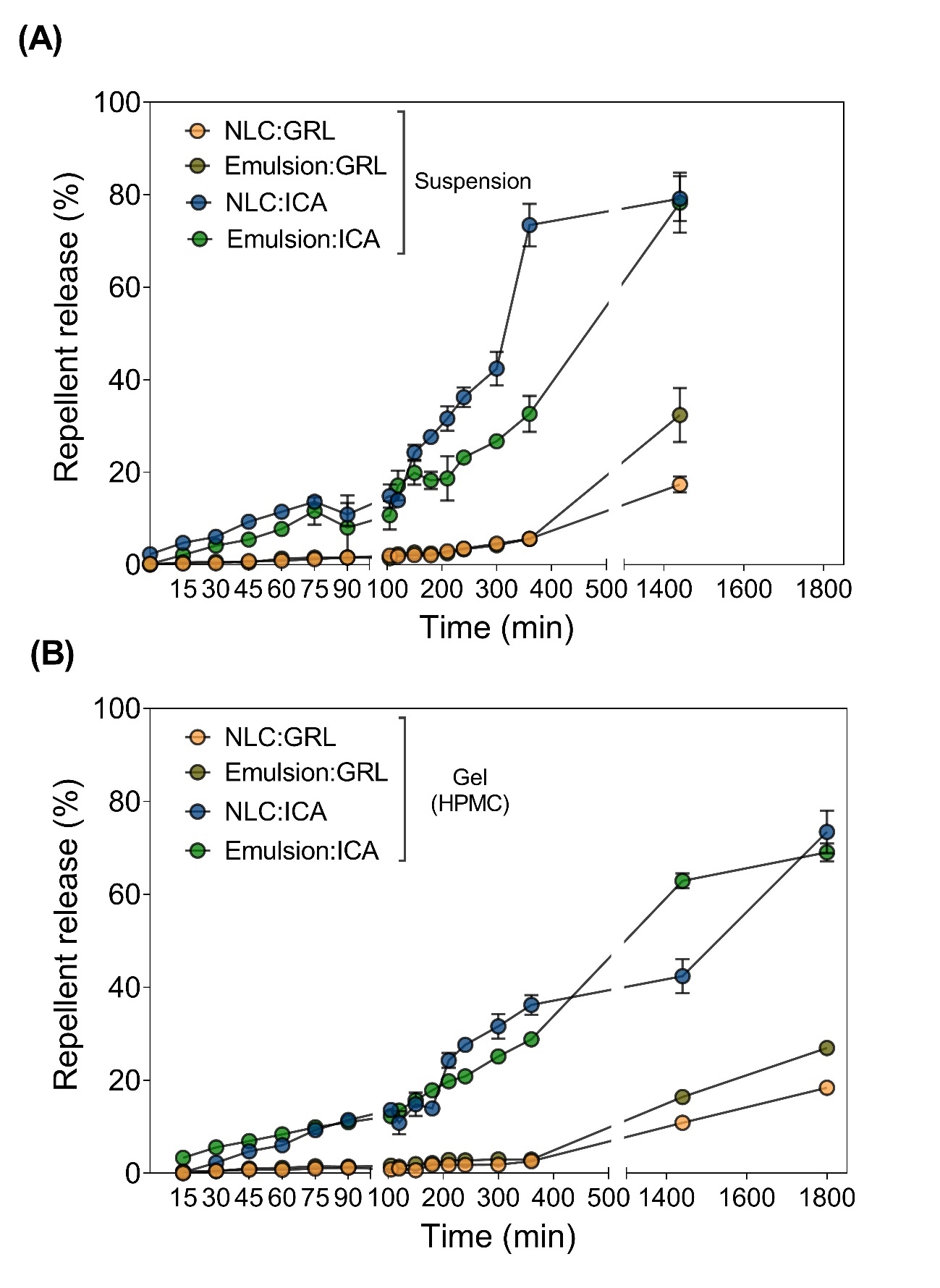


**Supplementary Figure 7.** Comparative release profiles for the repellents in the emulsion and NLCs formulations in the forms of A) suspension and B) hydrogel. The emulsion and NLCs contained geraniol (GRL) at 2% and icaridin (ICA) at 5%. The system was maintained under magnetic stirring (300 rpm), at 32.5 °C, for 32 h, under sink conditions. The analyses were performed in triplicate (values shown as mean ± SD).


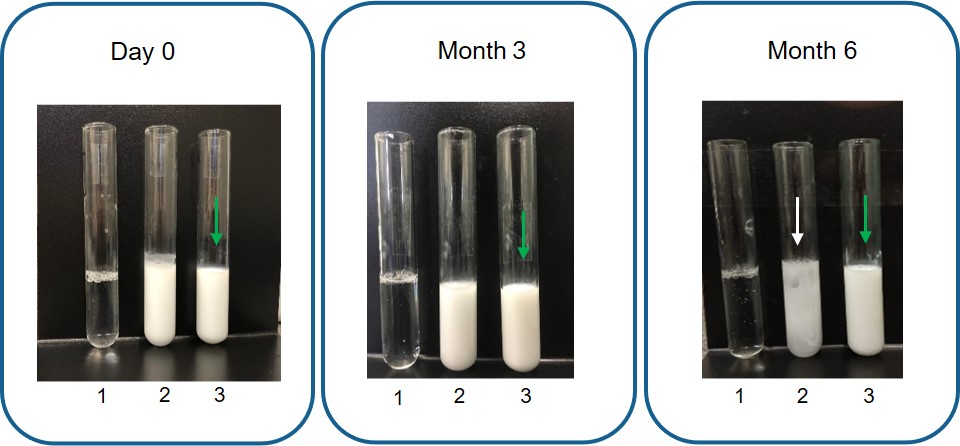


**Supplementary Figure 8.** Analysis of the organoleptic characteristics of the formulations over time (on day 0 and after 3 and 6 months): 1) control gel (2% HPMC); 2) emulsion containing 2% GRL and 5% ICA, incorporated in gel. 3) NCLs containing 2% GRL and 5% ICA, incorporated in gel.

**Tables**

**Supplementary Table 1.** Temperatures (T) and enthalpy variations (ΔHf) obtained from differential scanning calorimetry (DSC) analyses of the NLC main matrix (tripalmitin), the NLCs formulation without active ingredients (NLC empty), and the formulations of NLCs loaded with 2% GRL and 5% ICA, incorporated in suspension and gel. The system was heated from 10 to 250 °C, at a rate of 10 °C/min.


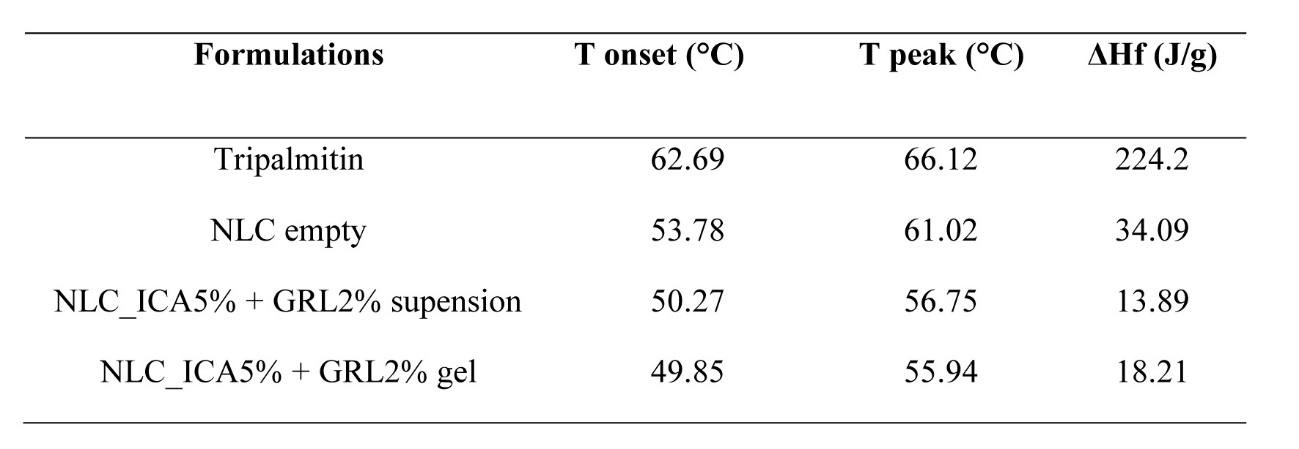


**Supplementary Table 2**. Parameter values obtained by applying mathematical models for the release of geraniol and icaridin from the NLCs in suspension and gel.


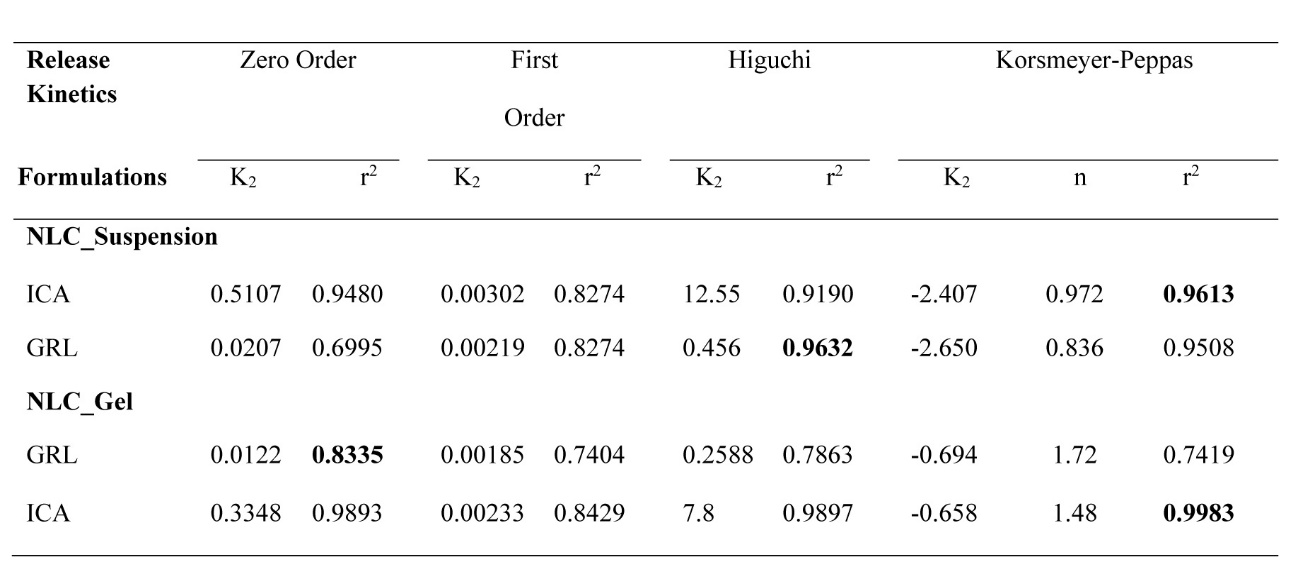


**Supplementary Table 3.** Measurements of pH during the accelerated stability testing up to 6 months, for the control gel (2% HPMC), the gel with emulsion containing 2% GRL and 5% ICA, and the gel with NLCs containing 2% GRL and 5% ICA. The formulations were kept under conditions of controlled temperature (40 ± 2 °C) and relative humidity (75 ± 5%).


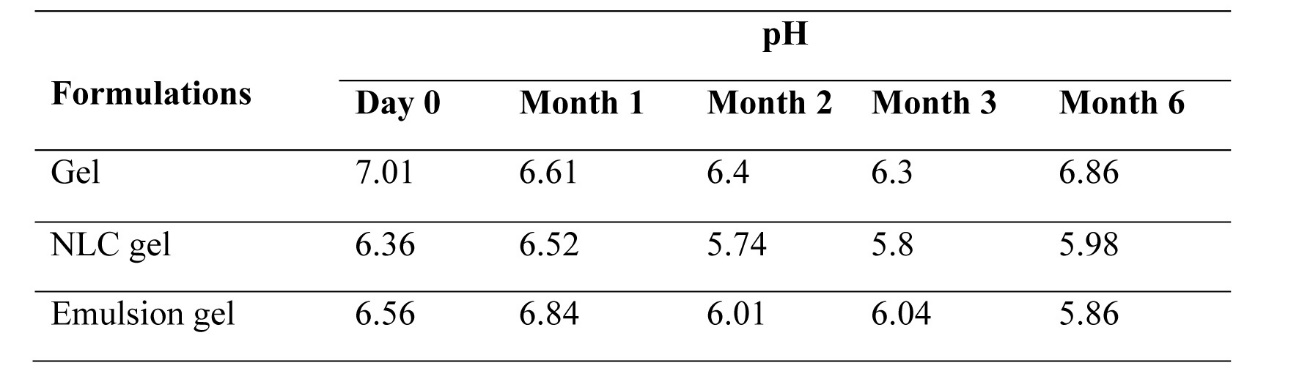


**Supplementary Table 4.** Measurements of density during the accelerated stability testing up to 6 months, for the control gel (2% HPMC), the gel with emulsion contain 2% GRL and 5% ICA, and the gel with NLCs containing 2% GRL and 5% ICA. The formulations were kept under conditions of controlled temperature (40 ± 2 °C) and relative humidity (75 ± 5%).


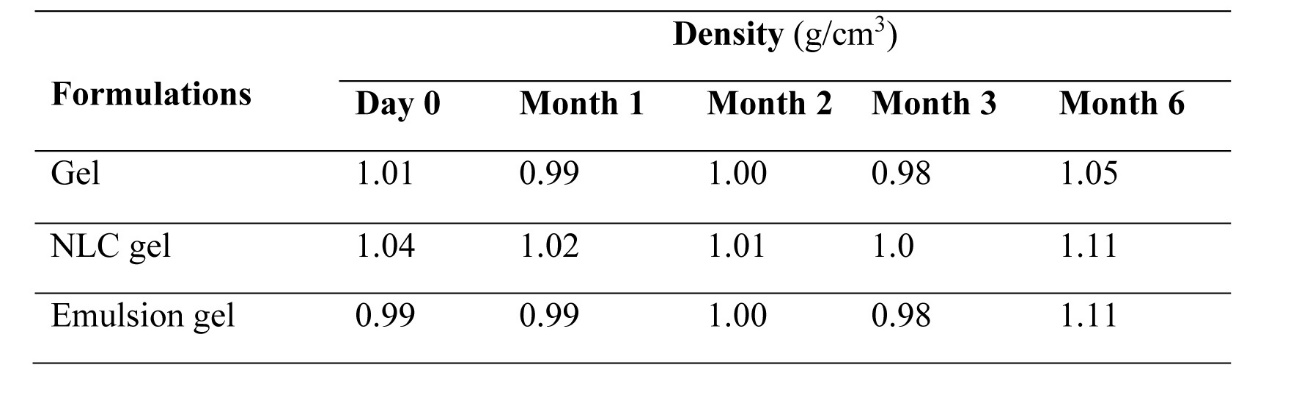


**Supplementary Table 5.** Microbial population analysis after 6 months under stability testing conditions. The formulations were kept under conditions of controlled temperature (40 ± 2 °C) and relative humidity (75 ± 5%).


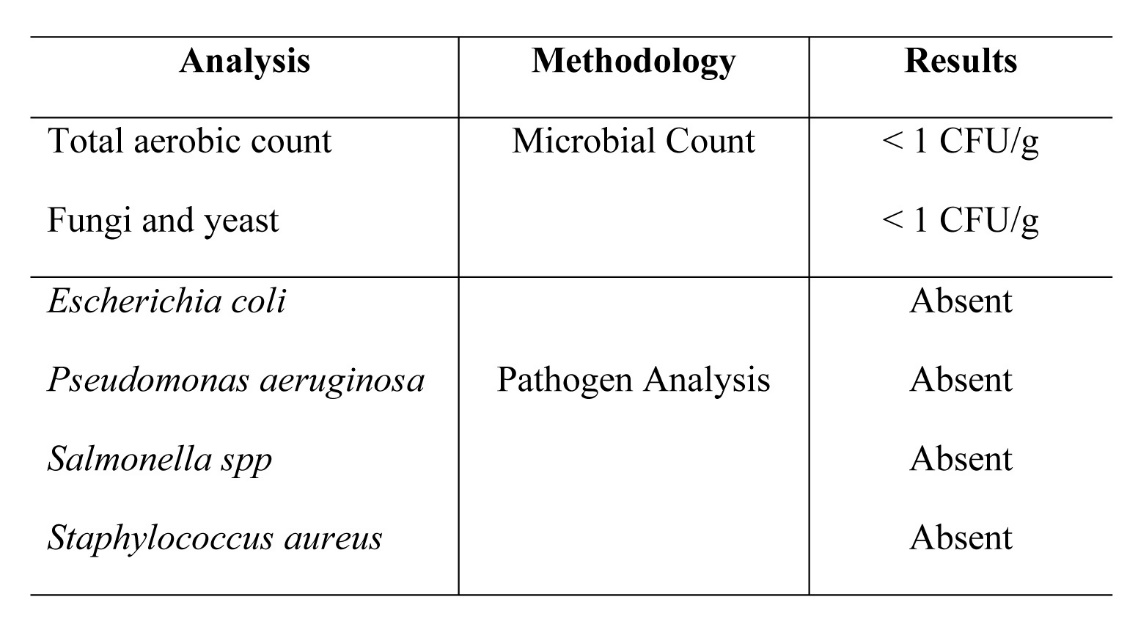

Supplement: Supplementary file 1 [file DataSheet1.docx]
